# Meta-analysis of the effects of smooth endoplasmic reticulum aggregation on birth outcome

**DOI:** 10.1186/s12884-021-03850-1

**Published:** 2021-05-12

**Authors:** Hongqin Zhang, Wenhui Hu, Ying Zhong, Zhenhua Guo

**Affiliations:** 1 Chengdu Jinjing Maternal and Child Health Hospital, Jinxin Research Institute for Reproductive Medicine and Genetics, No. 3 Sanguantang Road, Chengdu, 610051 People’s Republic of China; 2 Chengdu Xi’nan Gynecology Hospital of Jinxin Medical Group, No. 66 Bisheng Road, Chengdu, 610023 People’s Republic of China; 3 Key Laboratory of Combining Farming and Animal Husbandry, Ministry of Agriculture and Rural Affairs, Heilongjiang Academy of Agricultural Sciences, Animal Husbandry Research Institute, No. 368 Xuefu Road, Harbin, 150086 People’s Republic of China

**Keywords:** Aggregation, Births, Oocyte, Smooth endoplasmic reticulum (SER)

## Abstract

**Background:**

Smooth endoplasmic reticulum aggregation (SERa, SER+) has been reported to increase the risk of birth malformations and other abnormal outcomes, miscarriage, and perinatal complications. Other studies, however, suggest that SER+ embryos may develop into healthy infants. One report indicates that 25% of in vitro fertilization (IVF) centers discard SER+ oocytes. Thus, we investigated the effect of SER+ on birth outcomes in IVF and intracytoplasmic sperm injection.

**Methods:**

We performed a literature search using PubMed, ScienceDirect, Cochrane, Embase, Ovid, and Scopus. We found a total of 1500 relevant studies between 1978 and 2020 and conducted a meta-analysis to study the effects of SER+ on live births, birth weight, and the number of metaphase II (MII) oocytes retrieved per cycle.

**Results:**

Eleven eligible studies were included. If the SER+ zygote was evaluated again at the embryo transfer (ET) stage, SER+ did not affect birth or infant body weight. Stimulated ovaries producing too many oocytes per cycle were positively correlated with SER+ (OR = 1.28, 95% CI = 0.41–2.15; *p* = 0.004). SER+ was positively correlated with oocyte maturation rate, and observed heterogeneity in a previous meta-analysis was likely due to maternal age. Our data also showed that SER+ cycles produced more oocytes but achieved the same number of births from ET.

**Conclusions:**

The use of SER+ MII oocytes is rare, with the collection of many oocytes in 1 cycle potentially inducing SER+. SER+ may be more common than we originally thought, as some SER+ is found in all oocytes. Although SER+ positively affected oocyte maturation rate, it did not affect births. We hypothesized that this is because the best embryos are chosen at every step of the process, and the oocytes with the poorest characteristics are removed. We therefore suggest a standard method for measuring SER+. Although embryos produced from SER+ cycles can be used, they should only be transferred when no other suitable embryos are available over several cycles.

**Supplementary Information:**

The online version contains supplementary material available at 10.1186/s12884-021-03850-1.

## Background

Clinical studies have indicated that smooth endoplasmic reticulum aggregation (SERa or SER+) may increase the risk of birth malformation or other abnormal outcomes [1–5], miscarriage [2], and perinatal complications [3]. However, SER+ is quite common. During in vitro fertilization (IVF), SER+ appears in 10% of ovulation-induction cycles and in 19–34% of oocytes [6]. It is recommended to avoid SER+ embryos entirely [7, 8] and advised to also measure SER+ size [2]. Embryologic research has revealed that the smooth endoplasmic reticulum (SER) regulates early embryonic development via energy accumulation [4] and plays a key role in calcium storage and release [9]. SER+ also augments the oocyte maturation rate [10] and diminishes the fertilization rate [11]. Researchers have even reported the occurrence of complex chromosomal rearrangements with consistent 2q31 deletions [5].

Two systematic reviews undertaken in 2014 and 2019 concluded that SER+ embryos can be used when embryos of sufficient quality are not available [6, 9]. Some studies have suggested that SER+ embryos can develop into healthy infants after embryo transfer [10, 12–15]. Despite this, another study implied that 25% of IVF centers discard SER+ oocytes prior to intracytoplasmic sperm injection (ICSI) [16]. Thus, we performed a meta-analysis to study the effect of SER+ on live births, birth weight, and the number of metaphase-II (MII) oocytes per cycle.

## Methods

### Database search and screening studies

**Fig. 1:**
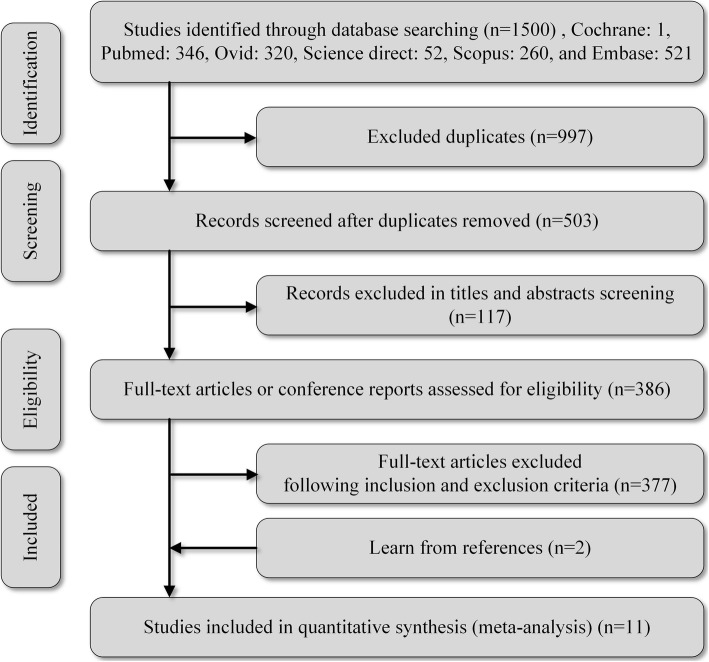
Summary of study selection. A total of 1500 studies were searched, 997 duplicate articles were removed, and 117 reports that were not related were excluded according to the title and abstract. After reading 386 studies, two additional articles were uncovered from the references. Finally, 11 studies were selected

Two authors (HQZ and WHH) independently conducted a literature search using PubMed (Medline), ScienceDirect, Cochrane, Embase, Ovid, and Scopus. Keywords used to search PubMed were endoplasmic reticulum AND (aggregation OR aggregate OR aggregates OR cluster OR clusters) AND (oocyte OR oocytes OR zygote OR zygotes OR embryo OR embryos) AND (“1978/01/01”[PDAT]: “2020/08/31”[PDAT]). The two authors then independently decided whether an article was to be assessed or not according to our study-eligibility criteria shown in Fig. 1 and Table 1. We did not specify randomized clinical trials (RCTs), as most of the included literature did not mention them.

**Table 1:** Inclusion and exclusion criteria

Inclusion	Exclusion
Species evaluated must include but are not limited to humans (clinical study)	Human oocytes not used
English	Non-English literature
SER+ of oocytes but not limited to SER+	No SER+ oocytes
IVF or ICSI for fertilization	Only embryo transfer
Births reported	No live births
SER+ and SER- contrasted	Lacking SER+ or SER-
Original research	Review

### Data extraction

Data extraction and assessment of risk bias were performed according to both Sargeant [17] and O’Connor [18]. Briefly, after appropriate data extraction, another author re-evaluated the details. When there was disagreement, a third author (YZ) was brought in to help establish a consensus. Potential bias was assessed by visual inspection of Begg’s funnel plots, and by using Egger’s linear regression [19] and Begg’s rank correlation tests. We performed statistical analyses using Stata 12.0 (Stata Corp, College Station, TX, USA), and *p* < 0.05 was considered to be statistically significant. A SER+ cycle was designated as 1 cycle in which one or more oocytes were SER+. We defined a SER+ MII oocyte as an oocyte that was observed to be SER+ and that developed to the MII stage [13].

### Meta-analysis

According to the type of raw data extracted, we used a continuous method to calculate the SER+ MII oocyte rate and infant body weight at birth. Effects of SER+ cycles and of SER+ MII oocytes on the number of births were analyzed with a dichotomous method. We assessed the SER+ cycle effect on births, specifically calculating births from MII oocyte number and embryo transfer (ET) number. To understand how SER+ and ovum pick up (OPU) number were related, we assessed the number of MII oocytes per cycle and noted the effect of SER+ cycles on birth weight. Heterogeneity was defined using a Higgins statistic, a *p*-value, and an *I*^2^ statistic (*I*^2^ > 50% indicated high heterogeneity) in a previous meta-analysis [20]. We used a fixed-effects analysis in the absence of heterogeneity and a random-effects analysis in the presence of heterogeneity, with subgroups in meta-analysis.

Births were defined as the number of new births divided by the number of ETs. SER+ cycles included at least one MII oocyte [13]. We carried out data analysis using Review Manager (Copenhagen: Nordic Cochrane Centre, Cochrane Collaboration, Version 5.4) for meta-analysis.

## Results

**Fig. 2:**
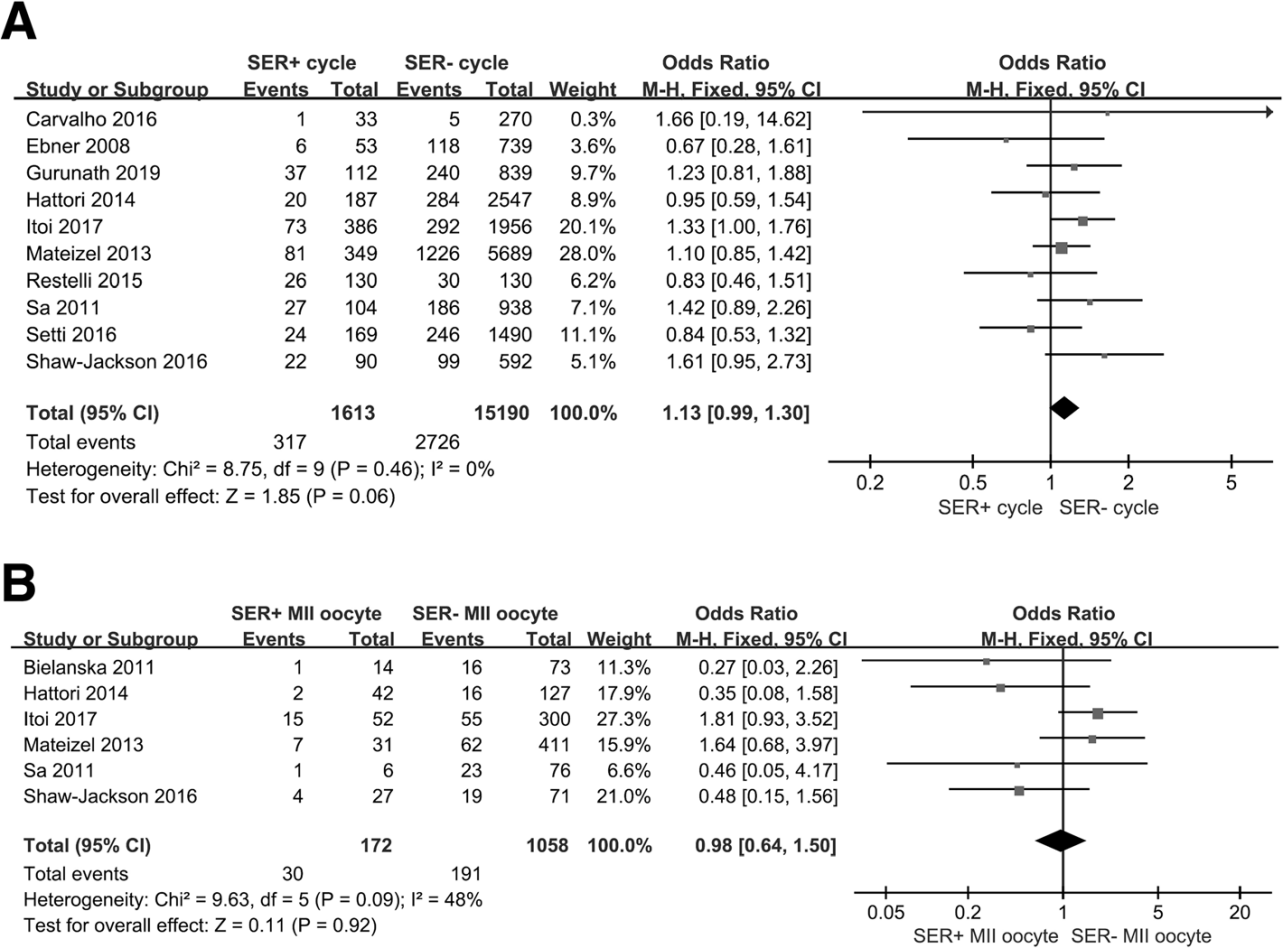
Forest plot of SER and births. **a**. Effect of SER+ cycles on births. Analysis of SER+ cycle and SER− cycle groups. There was no effect on birth. **b**. SER MII oocyte effect on births. Analysis of SER+ MII oocytes and SER− MII oocyte groups. There was no effect on birth. Total means of ET numbers, with the calculation based upon ET embryo number. CI = 95% confidence interval

**Fig. 3:**
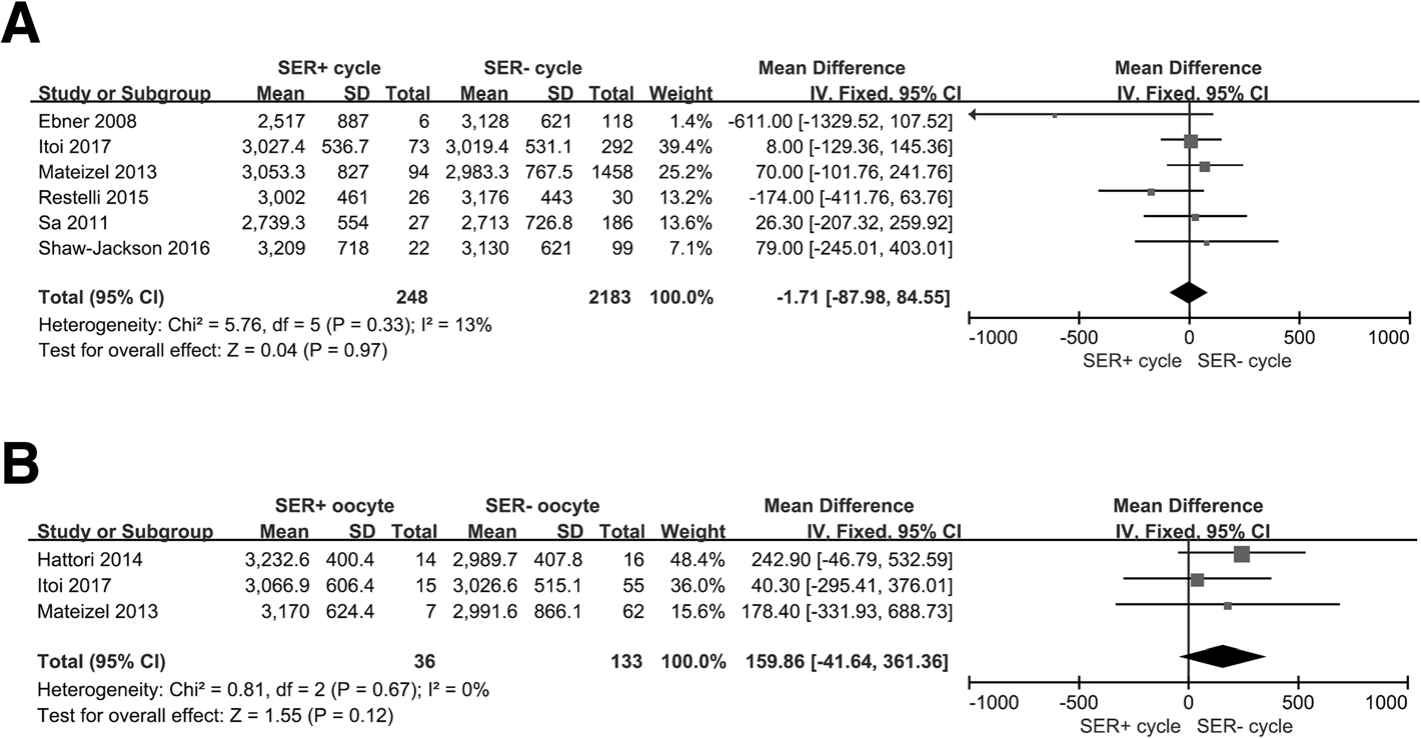
Forest plot of SER effect on birth weight. **a**. SER cycle effect on birth weight. Analysis of SER+ cycle and SER− cycle groups. There was no effect on birth weight. **b**. SER MII oocyte effect on birth weight. Analysis of SER+ MII oocyte and SER− MII oocyte groups. There was no effect on birth weight. Total indicates the birth number. CI = 95% confidence interval

**Fig. 4:**
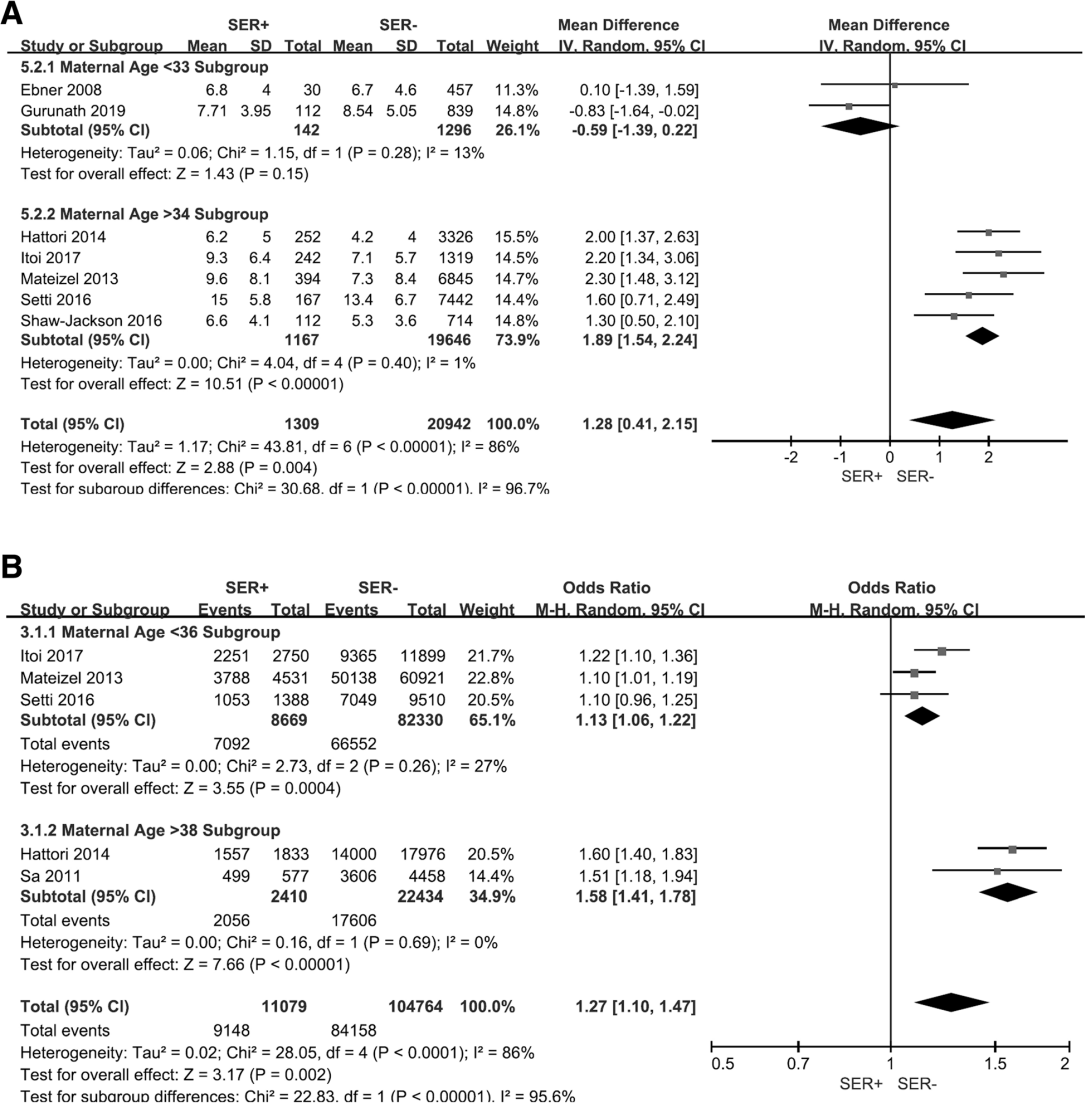
Forest plot of effect of MII oocytes per cycle on SER. **a**. Analysis of SER+ cycle and SER− cycle groups. SER+ cycles produced more MII oocytes. Stimulated ovaries producing too many oocytes per cycle were positively correlated with SER+. **b**. Analysis of SER+ MII oocyte-maturation rate. SER+ was positively correlated with oocyte maturation rate. CI = 95% confidence interval

We ultimately included in our meta-analysis 11 eligible studies that were published less than 12 years ago (Table 2) [3, 4, 10, 12–15, 21–24], and we noted that ovarian stimulation protocols were very common among them. We analyzed births and SER+ cycles (Fig. 2a) and SER MII oocytes (Fig. 2b), and observed no differences between SER+ (OR = 1.13, 95% CI = 0.99–1.3; *p* = 0.35) and SER− cycle/MII oocytes (OR = 0.98, 95% CI = 0.64–1.5; *p* = 0.13). Results were also similar with respect to birth weights (Fig. 3a and b). We uncovered no association between SER+ cycle/MII oocytes (OR = -1.71, 95% CI = -87.98–84.55; *p* = 0.33) and infant birth weight (OR = 159.86, 95% CI = -41.36–361.36; *p* = 0.67). Figure 4a shows that SER+ cycles generated more MII oocytes, and we posit that hormonally stimulated ovaries appear to produce too many oocytes per cycle and induce SER+ (OR = 1.28, 95% CI = 0.41–2.15; *p* = 0.004). SER+ also had a positive correlation with oocyte maturation rate (OR = 1.27, 95% CI = 1.1–1.47; *p* = 0.002; Fig. 4b). The heterogeneity we observed appeared to be due to maternal age (Fig. 4a and b).

**Table 2:** Characteristics of studies included in the review

No.	Studies	Year	IVF & ICSI	Maturation time (h) after hCG/GnRH	Ovarian stimulation	Embryo culture	ET^a^ time	Type	Maternal age (y)
1	Bielanska^b^	2011	ICSI	NT	NT	NT	Day 3 or 6	Vitrification and fresh	NT
2	Carvalho^b^	2016	ICSI	use hCG	Stimulation	NT	NT	fresh	NT
3	Ebner	2008	IVF & ICSI	36 (hCG)	Natural/Stimulated	Incubator	Day 3 or 5	Fresh	32.85 ± 5.05
4	Gurunath	2019	ICSI	35 (hCG)	Stimulation	NT	Day 3 or 5	Fresh	31.5
5	Hattori	2014	ICSI	36 (hCG/GnRH)	Stimulation	Incubator	Day 2 or 3	Vitrified and fresh	38.2 ± 4.7
6	Itoi	2017	IVF & ICSI	35 (hCG/GnRH)	Stimulation	Time-lapse live embryo imaging	Day 5 or 6	Vitrified and fresh	35.5 ± 4.4
7	Mateizel	2013	ICSI	36 (hCG)	Natural/Stimulated	Incubator	Day 3 or 5	Fresh	34.75 ± 0.15
8	Restelli	2015	IVF & ICSI	36 (hCG)	Stimulation	Incubator	Day 2–3 or 5	Vitrified and fresh	36.55 ± 3.65
9	Sa	2011	ICSI	36 (hCG)	Stimulation	Incubator	Day 2–5	Fresh	38.23
10	Setti	2016	ICSI	36 (hCG)	Stimulation	Incubator	Day 5	Fresh	34.3 ± 4
11	Shaw-Jackson	2016	ICSI	34–36 (hCG)	Stimulation	Incubator	Day 2–3 or 5	Fresh	35.65 ± 5.4

*NT* Not mentioned

^a^Embryo transfer

^b^Summary of the meeting: We tried to contact the authors of the two abstracts but failed, as there was not enough author information

**Fig. 5:**
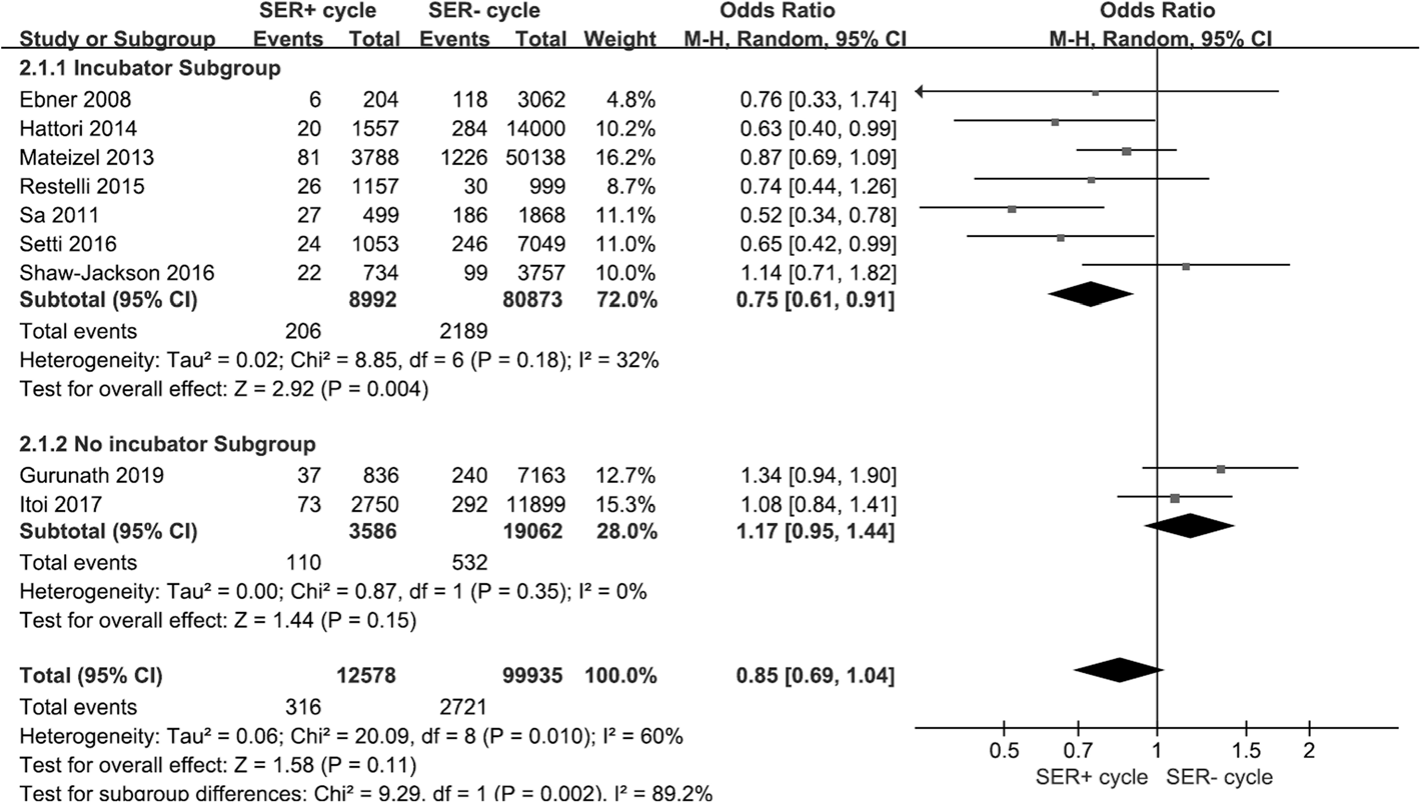
Forest plot of MII-oocyte use. Total indicates the number of MII oocytes. Analysis of SER+ cycle and SER− cycle groups. No statistical differences were observed between groups, with the calculation based upon MII-oocyte number. CI = 95% confidence interval

Our data indicated that SER+ cycles produced more oocytes but achieved the same number of births after ET, so many oocytes were wasted. Thus, we defined MII-oocyte use as the number of new births divided by the number of MII oocytes inseminated and compared SER+ and SER− cycles. Figure 5 shows that the SER+ cycle (2.51%, 316/12,578) data were not statistically different from SER− cycle groups (2.72%, 2721/99,935) according to MII-oocyte use. We did, however, observe heterogeneity due to using different embryonic culture incubators. Supplemental Fig. 1 shows funnel plots of MII-oocyte use relative to birth; there was no potential bias, and this lack of potential bias was also corroborated by Egger’s (*P* = 0.263) and Begg’s tests (Pr > |z| = 1).

## Discussion

### Primary finding

SER+ did not affect birth or infant body weight. Although ovarian stimulation protocols produced an overabundance of oocytes per cycle, and in our study caused SER+, we interestingly retrieved more MII oocytes from SER+ cycles, and SER+ was positively correlated with oocyte maturation rate.

In normal oocytes, SER manifests as multiple scattered spherical aggregates surrounded by mitochondria [25], whereas the phenomenon of SER+ shows aggregations in a single pattern [26]. SER+ may exist normally in MII oocytes and then disappear prior to pronuclear formation [2], and SER+ may also be present in unfertilized oocytes, degenerated oocytes, and embryos [21].

How SER+ occurs remains unclear, although studies suggest that it may originate from genetic abnormalities. SER+ can occur repeatedly in multiple ICSI cycles in the same patient, which has been explained by genetic factors [11, 27]. Intriguingly, studies have shown that SER+ is associated with elevated anti-Müllerian hormone (AMH) [28]. SER+ in the cytoplasm of oocytes is also correlated with E2 on the hCG-treatment day: the higher the peripheral blood E2 concentration, the more likely it is for SER+ to materialize [28]. Oocyte SER+ may also be caused by overstimulation of the ovaries [29]; for example, increasing GnRH and prolonging the duration of ovarian stimulation increases SER+ [3, 6, 30]. Maternal age and FSH dose are unrelated to the appearance of SER+, although follicle stimulation and oocyte collection significantly increase its risk [10]. Thus, the collection of numerous oocytes in 1 cycle might induce SER+.

Some investigators have reported negative biologic impacts of SER+, including a higher frequency of aberrant spindle formation and an elevated incidence of cytokinesis failure in embryos derived from SER+ oocytes [31]. We demonstrated that SER+ had a positive effect on oocyte maturation rate but did not affect births. We hypothesize that this is because the best embryos are chosen at each step in the process, and the most poor-quality oocytes (including those designated as showing SER+) are removed.

As we were not able to observe SER+ in oocytes under light microscopy, we turned to electron microscopy and noted aggregates of 2–9 μm [2]. The results suggested that SER+ is more common than previously noted due to its low visibility under light microscopy.

Oligonucleotide microarray analysis of SER+ oocytes versus SER− oocytes showed six down-regulated genes (*CROCC, FDXR, HAUS8, MAP 2, MRPL11,* and *RPS3*) and three up-regulated genes (*GPSM1, GPSM3,* and *RAP1GAP*) [32]. The gene-expression changes were determined to be involved in (i) cell and mitotic/meiotic nuclear division, (ii) organization of cytoskeleton and microtubules, and (iii) mitochondrial structure and activity [32]. None of these changes were linked to ER stress.

## Limitations

For this study, data were collected between January 1, 1978, and August 31, 2020—with a SER cycle having one or several SER oocytes and the remaining oocyte(s) classified as normal [4]. Since SER+ embryos are not the typically preferred embryos for transplantation, many of them are not transferred [3]. In addition, when SER+ MII oocytes are used in a grouping experiment, they are evaluated again before ET [21]; thus, the significance of this study may be limited.

A study in which the outcomes of SER+ cycles/oocytes were assessed indicated that fetal malformations with SER+ cycles were greater than for SER− cycles [6]. Therefore, although the SER+ cycle can be recovered after embryo transfer, the fetus may show congenital defects, and this should be considered when transplanting SER+ cycle embryos.

## Conclusions

We agree with the recent Alpha/ESHRE consensus [1] that transplantation of embryos with SER+ should be carefully considered. Embryos produced from SER+ cycles can be used but should only be transferred if no other suitable embryos are available over several cycles. This may provide the only prospect for completing a pregnancy to term with the birth of a healthy baby, and we feel this is a choice patients should have the right to make. The present technology requires patient consent because the patient must be made aware of the risk of complex chromosomal rearrangements with consistent 2q31 deletions.

## Supplementary Information

**Additional file 1: Supplemental Figure 1.** Funnel plots of MII-oocyte use. This study was symmetrically distributed about the funnel plots, showing no potential bias.

## Data Availability

We declare that the materials described in the manuscript, including all relevant raw data, will be freely available to any scientist wishing to use them for non-commercial purposes, without breaching participant confidentiality.

## Abbreviations

SER: Smooth endoplasmic reticulum aggregation
IVF: In vitro fertilization
MII: Metaphase II
ET: Embryo transfer
ICSI: Intracytoplasmic sperm injection
OPU: Ovum pick up
AMH: Anti-Müllerian hormone
