# Supplementary figures and images for: Meta-analysis of the effects of smooth endoplasmic reticulum aggregation on birth outcome
Source: BMC Pregnancy Childbirth. 2021 May 12;21:374. doi: 10.1186/s12884-021-03850-1 (PMC8117493; doi:10.1186/s12884-021-03850-1)

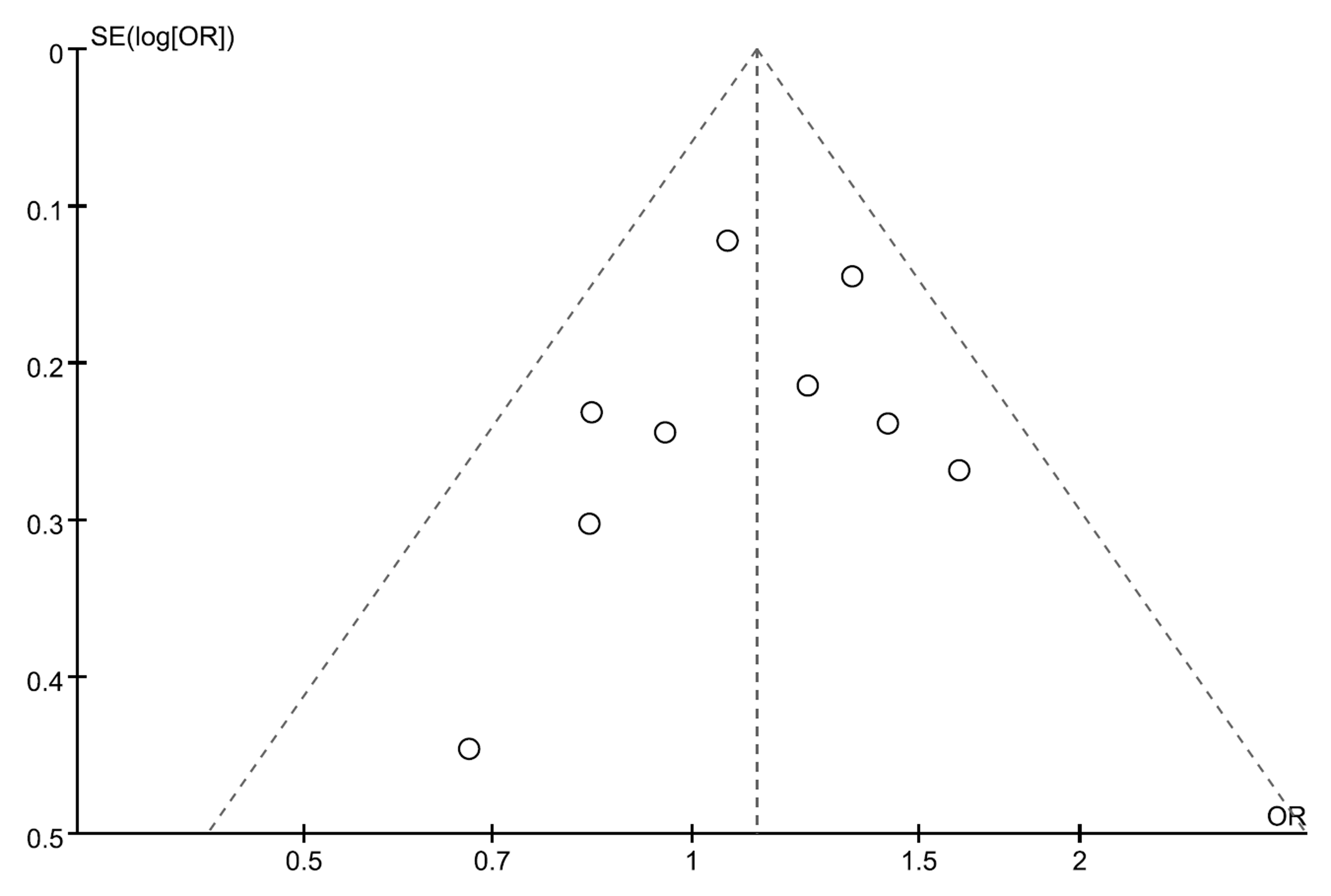

Supplement: Supplementary file 1 — Additional file 1: Supplemental Figure 1. Funnel plots of MII-oocyte use. This study was symmetrically distributed about the funnel plots, showing no potential bias. [file 12884_2021_3850_MOESM1_ESM.jpg]
